# Cavernous Sinus and Generalized Venous Thrombosis Following Rhinoplasty in a Young Patient

**Published:** 2012-07

**Authors:** Mohammad Motamed Shariati, Ahmad Meymane Jahromi

**Affiliations:** 1 Department of Plastic Surgery, Mashhad University of Medical Sciences, Ghaem Hospital, Mashhad, Iran; 2 Department of Otolarhngology, Mashhad University of Medical Sciences, Imam Reza Hospital, Mashhad, Iran

**Keywords:** Rhinoplasty, Thromboambolism, Venous thrombosis

## Abstract

Venous thromboembolism at lower age groups and in the absence of other known risk factors should raise doubt of underlying genetic disorders and thombophilia. It is discovered unexpectedly in various medical procedures and may cause severe complications and life threatening problems.

In the current study, potentially life threatening complications of a genetic disorder in a previously healthy case is discussed. The important point in this case was delay onset of complication and its severity when was unexpectedly discovered. This situation may happen in almost every cosmetic operation and when the surgeon is not well prepared for managing this medical condition that it may lead to a dramatically poor prognosis for the patient.

## INTRODUCTION

The attention paid by plastic surgeons to patient’s coagulation status has two contradictory aspects. On one hand, in cosmetic surgery of the face, eyelid and nose, plastic surgeons are concerned about clotting problems such as tendency to bleed. Subsequently, list of contraindicated medicines that interact with normal coagulation, is rising in this type of surgery. On the other hand, in patients undergoing body surgery, the probability of deep vein thrombosis (DVT) and pulmonary thromboembolism (PTE) is a major concern to both the surgeon and the patient before the operation.^1^

This concern arises from the fact that mortality of postoperative PTE has the potential of being prevented in most case. In this clinical issue, candidates for body surgery are considered as a high risk group for PTE. The role of thrombophilia in association with venous thromboembolism has been known for a long time.^2^,^3^ Another important point in this issue is the known role of thrombophilia and induced DVT in failure of free flaps.

Moreover, deficiency of anticoagulant factors (types C and S), anti-thrombin and genetic mutations may lead to thrombophilia as well. Hyperhomocysteinemia is another disease associated with an increased risk of thrombosis in arterial and venous systems.^4^-^6^ In this condition, cytosine is replaced with thymidine in the 677th nucleotide in the MTHFR region of the gene.^7^ The moderate form of the disease is more prevalent in most populations.

In this study, authors report the first case of septorhinoplasty which deals with consecutive thrombotic events in different parts of the venous system. In this patient, postoperative full panel blood screen detected an undiagnosed hereditary thrombophilia that was going to become life threatening for him.

## CASE REPORT

A 27 years old Iranian male was referred to the clinic as a candidate for septorhinoplasty surgery. After initial examinations and X-ray imaging, the following tests were requested for the patient including PT, PTT, INR, BT, CT CBC and platelet count. All results were reported as normal. Surgery was carried out by open approach and septal harvest and external osteotomy. The patient was discharged after removal of the nasal pouch of antibiotic as well as pain killer prescription a day after surgery. At the end of the first week, the splint was removed; the patient’s general condition was fine and had no major complaints, so his next visit was scheduled for two weeks later.

**Fig 1:**
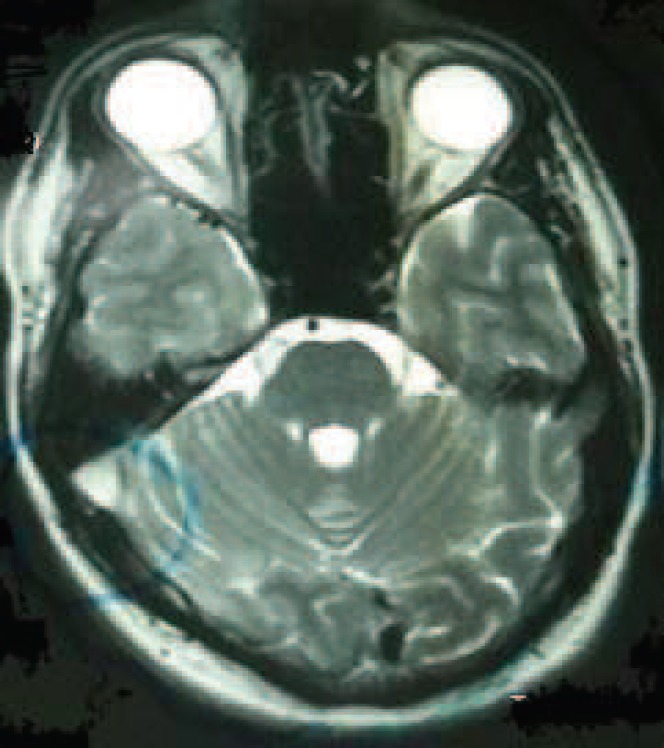
Axial brain MRI in T2-weighted sequence showing abnormal signals in sigmoid sinus.

**Fig 2:**
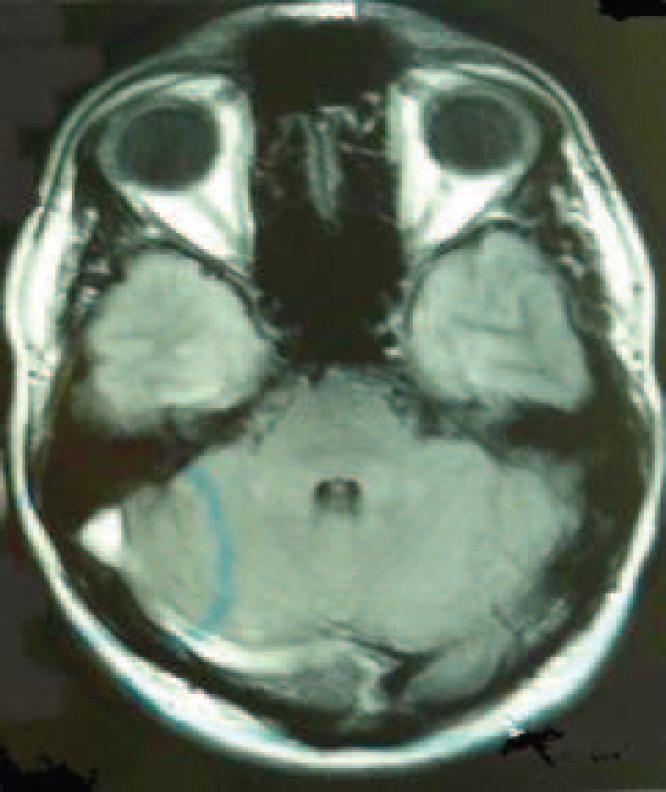
Axial brain MRI in FLAIR sequence showing abnormal signals in sigmoid sinus.

On the second visit, the patient had complaint of headache and he was asked to return if it continued despite the medications. During the next two days, his headache worsened and the patient was hospitalized. The patient was afebrile and conscious but neck rigidity was found in physical examination. Funduscopy was normal and there was no diplopia or eye movement impairment. Neurologic consultation was done with brain CT scan and MR angiography (Figure 1 and 2) that revealed diffuse brain edema, cavernous and sigmoid sinus thrombosis.

**Fig. 3:**
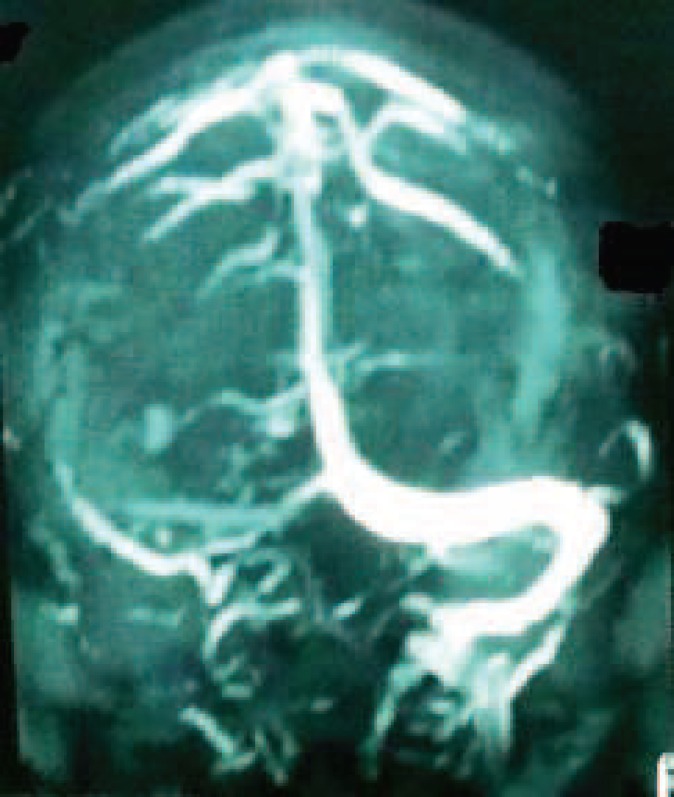
Brain MRV, sigmoid and lateral sinus in right side is not seen due to thrombosis.

Then anticoagulant therapy was initiated. Then he developed seizure which was controlled by diazepam and dilantin. Three days after admission, his seizure was fully controlled; however he developed pain and edema in both lower extremities. Doppler sonography revealed extensive thrombosis in both ileofemoral venous systems (Figure 3).

Consultation with cardiologic, hematologic and vascular surgery services were performed. Considering his condition, a filter was placed in his inferior vena cava in one step, while in at the same time; laboratory studies including proteins C and S deficiency assay were done which were reported normal. The studies on gene mutation demonstrated the heterozygote type of MTHFR C677T mutation. His signs and symptoms improved gradually and he was discharged after administration of coumadin and PT control tests after 8 days.

## DISCUSSION

Venous thromboembolism at lower age groups and in the absence of other known risk factors should raise possibility of an underlying genetic disorder. In fact, thrombophilia is an example of the same hereditary diseases, predisposing the patient towards venous thromboembolism.^8^ Thrombophilic genetic diseases are divided into two categories including mutations in DNA which result in deficiency in a blood anticoagulant factor. Deficiencies of proteins C and S and also anti-thrombin III fall into this category. In the second group, polymorphism occurs in a single nucleotide. The most common examples of this group are factor V Leidin and C677TMTHFR mutation.

Increased level of homocysteine is a risk factor for vascular thrombotic diseases. High level of homocystein in circulation, inhibits prostacyclin synthesis. Active factor V inhibits activation of protein C and down-regulation of thrombomodulin expression and block tPA binding to endothelial cells. All mentioned above are procoagolant activities that can lead to excess thrombotic events. One of the most common genetic defects of homocystein metabolism is mutation in the enzyme methylene tetrahydrofolate reductase (MTHFR). This enzyme is responsible for conversion of 5, 10 methylene tetrahydrofolate to 5 tetrahydrofolate for synthesis of acid folic. One of the most common MTFHR mutations is a nucleotide transition from C to T at base position of 677. The mutation is common in Caucasian but vary in rate with a homozygous rate of five percent in Finish to 20 percent in French people.

The role of this kind of mutations in association with venous thromboembolism has been emphasized in some recent reports.^8^,^9^ In a survey conducted in Amman, in 35 percent of patients referred for the first thromboembolism attack, the same gene mutation was detected.^10^ In another study, investigating thrombophilia in 198 patients with venous thromboembolism, it was shown that factor V Leidin and prothrombin G20210A had a bigger role than MTHFRC677T in thrombose formation. It was shown that, in cases with more than one mutation, thrombophilia had a greater risk of thrombosis.^11^ Another study in Bahrain confirmed the role of mutation in this specific gene in stroke occurrence.^12^

The role of mutation of this gene has been approved in thrombosis of the mesenteric system.^13^,^14^ In another study, the role of gene mutation in relation to cerebral vein thrombosis was evaluated with the same risk in children and adult. Intracranial complications of rhinoplasty are uncommon but if happen are very troublesome. This is obvious that neither the patient nor the surgeon can cope with the occurrence of such events. Rhinorrhea, meningitis and cavernous sinus thrombosis were the most common intracranial complications.^17^-^21^

Carotid-cavernous fistula has also been reported in one case; while, the most frightening complication in this category was cavernous sinus thrombosis.^22^ Several points are discussable in this patient. First of all, this event occurred 17 days after the operation. On the other hand, rarity of such a problem caused a delay in diagnosis. So his signs and symptoms progressively propagated. Actually we were lucky that we saved his life and now he is doing well two years post operatively.

Recently, a study has reported the increasing attention paid by plastic surgeons to the coagulation status of the patients,^1^ mentioned that hereditary thrombophilia is a major con cern in body contouring patients, but rhinoplasty patients were not considered as a high risk groups.

There are two unresolved questions: 1- Is rhinoplasty a cosmetic procedure in a case of thrombophilia? If no, how we should prepare the patient for operation? 2- Can rhinoplasty be the cause and trigger point for progressive thrombophilia?
